# Adverse Childhood Experiences and Problematic Media Use: Perceptions of Caregivers of High-Risk Youth

**DOI:** 10.3390/ijerph18136725

**Published:** 2021-06-22

**Authors:** Sarah E. Domoff, Aubrey L. Borgen, Nicole Wilke, Amanda Hiles Howard

**Affiliations:** 1 Department of Psychology, Central Michigan University, Mount Pleasant, MI 48858, USA; borge1al@cmich.edu; 2 Applied Research and Best Practice Initiative, Christian Alliance for Orphans, Arequipa 04017, Cayma, Peru; nicole@cafo.org; 3 Department of Psychology, Samford University, Homewood, AL 35229, USA; ahoward6@samford.edu

**Keywords:** adverse childhood experiences, problematic media use, high-risk youth

## Abstract

Youth with a history of adverse childhood experiences (ACEs) are more likely to develop risky health behaviors. With the increase of media use in the general population, it is likely that these high-risk youth are developing maladaptive behaviors associated with media use (i.e., problematic media use). The goals of this article are (1) to describe symptoms of problematic media use in high-risk youth and (2) to determine whether ACEs are related to problematic media use in this population. Data were collected through online questionnaires from 348 parents or legal guardians of children ages 5 to 18 years, the majority of whom had been adopted. Parents and guardians reported on the child’s history of ACEs and completed the Problematic Media Use Measure-Short Form (PMUM-SF). Almost half of the participants reported that their child had a history of four or more ACEs (48.9%). Caregivers of foster or adopted children reported more symptoms of problematic media use than those reporting on their biological children. After adjusting for covariates, the number of ACEs predicted problematic media use above and beyond variance explained by demographic factors or screen time amount. Children with a history of ACEs had higher problematic media use compared to children without ACEs.

## 1. Introduction

The influx of mobile devices over the past decade has modified daily life across generations, with their presence affecting almost all domains of functioning. As individuals increasingly own multiple screen media devices, the amount of time spent on the devices also continues to grow. Ownership of smartphones has steeply increased over the past decade, rising from merely 35% of Americans owning a device in 2011 to 81% currently owning at least one smartphone [1]. Due to the convenience of these handheld media devices, 48% of young adults report that they are online constantly [2]. These effects are consistent for younger demographics as well: 53% of 11-year-olds own a smartphone, increasing to 69% by the age of 12 [3]. Over a fourth of adolescents use screen media for more than 8 h each day, while 15% of ‘tweens’ report similar levels of use [3]. This is a significant increase in use over the past decade: In 2016, adolescents spent at least twice as much time online compared to 2006 [4]. Furthermore, this pattern was consistent across socioeconomic status, race/ethnicity, and gender [4].

With this increase in device ownership and usage, problematic media use among youth has become a prevalent concern [5]. As a whole, problematic media use is defined as using devices in such a way that it interferes with important daily living activities [6]. Increased mobile device use has been found to decrease quantity and quality of sleep, frequency of physical activity, and academic performance [7,8]. When media use interferes with these important domains of child and adolescent life, it can result in poor mental health and symptomatic behaviors such as difficulty in stopping media use, frustration when not able to use screen media, and thoughts focusing on use of devices [6]. Despite a societal focus on decreasing hours spent with screens, these problematic media use behaviors are more predictive of psychosocial difficulties than amount of screen time [6].

The majority of research about problematic media use has focused on typically developing youth or convenience samples (indeed, most research on children’s media use relies on samples of typically developing children) [9]. However, research has identified a number of risk factors that are linked to problematic use. Being male and growing up in a single-parent household are both risk factors for developing video game addiction, while higher parent-adolescent conflict and overall family dysfunction are predictive of problematic internet use [10,11].

Similarly, another possible risk factor is a history of ACEs. ACEs, or adverse childhood experiences, are defined as traumatic events, such as sexual, physical, or emotional abuse, that can have negative and long-term effects on an individual’s well-being [12]. Research suggests that adolescents who have experienced a higher number of ACEs have poorer self-regulatory abilities compared to those who have experienced fewer [13]. Individuals who have experienced ACEs are more likely to exhibit a variety of mental health concerns, including depressive symptoms, antisocial behavior, and suicidal ideation/attempts [14]. In addition to psychological consequences, ACEs are also related to negative health behaviors, such as smoking, binge drinking, and the use of other substances [15]. Similarly, a recent study using a national sample of youth demonstrated that those experiencing more ACEs were at a higher risk for heavy digital media use [16]. Specifically, youth who were reported to experience four or more ACEs were at least three times more likely to also have high levels of media use reported by caregivers (more than four hours of use on a typical weekday) [16]. However, reported amount of time with media devices is only one aspect of problematic media use, and does not capture the nuances of how devices interfere with daily life [6].

The current study seeks to determine if, similar to other negative health behaviors, there is an association between ACEs and problematic media use. Research has demonstrated that maltreated youth are at a disproportionate risk for problematic internet use and problematic social media use; however, the study of problematic media use as a whole is lacking for this age group [17,18]. With adults, evidence has been found, albeit retrospectively, that ACEs correlate with problematic media use [19]. Based on prior research, we thus predict that greater ACEs will associate with greater problematic media use. While the current study does not investigate mediators of this association, previous research has identified mechanisms that support this hypothesis. Poor self-regulation has been found to be associated both with number of ACEs and increased problematic media use [13,20]. Other potential mechanisms associated with problematic media use include lack of household structure, poor child self-efficacy, and strained parent-child relationships [21]. Additionally, parenting stress has been found to mediate the association between ACEs and increased media use [16].

A second gap in the literature that our study seeks to address regards sample characteristics. As we have mentioned, most research focuses on typically developing youth or samples of convenience [9]. We know that children involved in foster care or child welfare systems are more likely to have ACEs, yet this high-risk population has rarely been represented in research on children’s media use [22]. As such, the goals of this article are (1) to describe the symptoms of problematic media use in high-risk youth and (2) to determine whether ACEs are related to problematic media use in this population. Exploring the links between adverse experiences during childhood and problematic media use could help inform intervention targets for agencies working with highly vulnerable youth.

## 2. Materials and Methods

From January 2019 through April 2019, recruitment notices were posted on the [blinded] institute’s (coalition connecting more than 200 non-governmental organizations serving vulnerable children and families) website, distributed by email through parent and professional networks, and emailed to potential participants on [blinded] institute’s distribution list. The recruitment information was further disseminated via snowball sampling. Parents could complete the questionnaires for any child between the ages of 5 and 18 years old who had been living with them for at least 6 months. All participants provided informed consent before completing the measures. The questionnaires were completed online and presented in random order. Surveys were taken via the Qualtrics website, with both desktop and smartphone versions available. Ethical approval was obtained from the author’s Institutional Review Board.

Participants were 348 parents/legal guardians. Demographics for both parent/legal guardians and children can be found in Table 1. Children ranged in age from 5 to 18 years (*M* = 11.94; *SD* = 4.11). Over half the children were male (53.3%). The majority of the children were White (50.3%) with a minority of children being Black/African American (21.1%), Latino/a (15.0%), and Asian (11.8%; see Table 1). Nearly a quarter (22.1%; *n* = 77) were biological children of the parent/legal guardian. The remaining 271 children (77.9%) were adopted. Of those children who joined their family through adoption, 42.2% (*n* = 147) resided in US foster care prior to adoption, 25.0% (*n* = 87) resided in a non-US residential facility, and 10.6% (*n* = 37) were in non-US foster care. For children with a history of adoption, average age at entry into care was 14.75 months (*SD* = 29.31) and average amount of time in care was 25.61 months (*SD* = 29.54). All children had been residing with their adoptive caregiver for at least six months.

The following demographic characteristics were assessed regarding the parent/guardian: age, gender (coded as 1 = male, 2 = female), and race/ethnicity. Parents/guardians reported on child age, gender (coded as 1 = male, 2 = female), race/ethnicity (coded as 1 = White, 2 = other race/ethnicity), and the child’s average weekly screen time. Additionally, for non-biological children, parents/legal guardians reported the child’s type of placement prior to adoption, age of child when he/she entered alternative care (i.e., foster care, institutional care, etc.), and length of time in alternative care prior to adoption (see Table 1).

Parents/guardians reported on whether their child experienced any of 10 adverse childhood experience categories, including abuse, neglect, and loss of caregiver [12]. Parents were asked to complete the ACEs questionnaire to the best of their ability, regarding their child’s history. Sample items include: “While your child was growing up, did they live with someone who had a substance abuse problem?” and “While your child was growing up, did they witness domestic violence (caregiver was pushed, grabbed, slapped)?” Pre-adoptive records for children may be incomplete, especially for children adopted internationally. See Table 1 for the frequency of ACEs experienced by children in this study. Approximately 49% of the sample had a history of four or more ACEs. See Table 2 for frequencies of different types of ACEs in this sample.

The Problematic Media Use Measure-Short Form (PMUM-SF) was used [6]. The PMUM-SF consists of nine items (α = 0.94) derived from criteria for Internet Gaming Disorder as specified in the DSM-5. Parents answered questions about the frequency of their child’s behavior on a 5-point Likert scale, ranging from ‘never’ to ‘always’. Sample items include: “My child becomes frustrated when he/she cannot use screen media” and “My child’s screen media use causes problems for the family.” The mean score for this measure was used for analyses, with higher scores indicating more problematic media use.

Descriptive statistics were calculated to provide average problematic media use scores by type of ACE. Bivariate correlations were then conducted to examine associations among ACEs, alternative care placement length, total weekly screen time, and problematic media use. Next, linear regression analyses were conducted, with Step 1 including the covariates of child age, child race/ethnicity, length of time in care, gender, and weekly screen time; in Step 2, number of ACEs were entered.

## 3. Results

As expected, the children in the current sample had experienced a high number of ACEs (M = 3.67; *SD* = 3.16) with nearly half (48.9%) reporting four or more. Parents reporting on biological children (*n* = 77) had lower PMUM levels (M = 2.28, *SD* = 0.83), compared to foster or adopted children (*n* = 271; M = 2.96, *SD* = 0.91; t (346) = −5.90, *p* < 0.01).

Mean PMUM levels by type of ACE were first examined. Across each type of ACE, children with the ACE had higher problematic media use scores compared to youth without the ACE (see Table 2). Based on bivariate correlations, greater ACEs and greater length of time in foster/institutional care were associated with higher problematic media use (r = 0.35, *p* < 0.01 and r = 0.30, *p* < 0.01, respectively). Adjusting for child age, child race/ethnicity, length of time in foster care, gender, and amount of children’s weekly screen time, we found that total ACEs predicted problematic media use (B = 0.24, *p* < 0.01), above and beyond variance explained by child demographic factors and weekly screen time (see Table 3).

## 4. Discussion

This study sought to examine problematic media use among high-risk youth and examine whether ACEs were associated with greater problematic media use. Children with any type of ACE had higher problematic media use compared to children without ACEs. Additionally, we found that ACEs emerged as a risk factor for problematic media use among high-risk youth, over and above important confounders. As such, vulnerable youth may be a population who should be targeted to receive assistance around managing media use, which may be interfering with their functioning.

Although mechanisms linking ACEs to problematic media use were not examined in this study, disrupted child self-regulation may account for the findings. A common risk factor for problematic media use is inhibited ability to self-regulate. As with other types of addiction, individuals with poor self-regulation skills may be more likely to develop internet gaming disorder [20]. It is possible that self-regulation difficulties may explain the association between ACEs and problematic media use. For example, prior research suggests that adolescents who have experienced greater ACEs have poorer self-regulatory abilities than those who have experienced fewer [13]. Future research on youth with histories of trauma and comorbid psychiatric conditions is strongly encouraged to replicate these findings and to examine the role of self-regulation as a mediator in a longitudinal research design.

Additionally, future research should address some of the limitations of this study. In particular, future research should examine dyadic processes and include multiple caregiver reports (e.g., both mothers and fathers, as well as other caregivers). Assessing adolescents’ perceptions of their own problematic use may also be informative. Examining the mechanisms of influence (i.e., self-regulation deficits related to early disrupted attachment as well as those proposed by Domoff et al.) is critical to inform intervention [21]. Including parental monitoring or other media parenting practices is also recommended as these variables may moderate the impact of ACEs on problematic media use. Finally, we suggest future research include covariates not measured in this study, such as family socioeconomic status and parental education.

Although there are limitations to this study (i.e., all parent report, retrospective accounting of child history of trauma, which may be limited given the few biological parents in this study, no objective measurement of screen time), a strength of this study was the recruitment of caregivers of youth with significant histories of trauma and involvement with child welfare systems. These youth are under-represented in the research and may be more at risk for problematic media use.

## 5. Conclusions

Overall, the findings suggest that problems with managing media use could be greater for youth with ACEs. Although preliminary, these findings do support querying about foster/adopted youths’ media use when providing care. Parents of these vulnerable youth may be a particular group for whom pediatricians should provide supports/guidance around media use, such as discussing a family media plan and recommending other online resources (e.g., Common Sense Media) [23].

Regarding clinical implications, our study supports screening youth with histories of adverse childhood experiences (or other early stressful life events) for problematic media use. In particular, the Problematic Media Use Measure (PMUM) and the Problematic Media Use Measure-Short Form (PMUM-SF) are brief screeners that mental health clinicians can use to identify whether vulnerable youth are exhibiting early signs of problematic media use [6]. In addition to dysregulated use, clinicians may seek to screen for other types of problematic use (e.g., online victimization or risky use) and media parenting practices to address in treatment. As access to digital media continues to grow (particularly during the pandemic), understanding how to promote safe and regulated media use will be critical [24].

## Figures and Tables

**Table 1:** Participant characteristics.

	*n* (%)/M (*SD*)
Child characteristics	
Age, years	11.94 (4.11)
Gender	
Female	161 (46.7%)
Male	184 (53.3%)
Race	
White	174 (50.3%)
Black	73 (21.1%)
Asian	41 (11.8%)
Latino/a	52 (15.0%)
Other	6 (1.8%)
Placement History	
Biological Parent	77 (22.1%)
US Foster Care	147 (42.2%)
Non-US Foster Care	37 (10.6%)
Non-US Residential Treatment	87 (25.0%)
Age of entry (months) to carefoster care, and hx of mental health conditions, # of ACEs	14.75 (29.31)
Length of time (months) in care	25.61 (29.54)
# of ACEs	3.67 (3.16)
Four or more ACEs	170 (48.9%)
Average weekly screen time	32.16 (31.56)
Average PMUM score	2.81 (0.94)
**Parent characteristics**	
Age, years	43.48 (8.84)
Gender	
Male	43 (12.4%)
Female	305 (87.6%)
Race	
White	335 (96.3%)
Black	5 (1.4%)
Asian	3 (0.9%)
Latino/a	3 (0.9%)
American Indian/Alaskan Native	2 (0.6%)

**Table 2:** Prevalence of ACEs and average problematic media use (PMU) by ACE history.

	*n* (%)	PMU (M/*SD*) with ACE	PMU (M/*SD*) no ACE	t (346)
Emotional abuse	167 (48)	3.07 (0.90)	2.58 (0.91)	−5.03 **
Physical abuse	154 (44)	3.11 (0.88)	2.58 (0.92)	−5.40 **
Sexual abuse	57 (16)	3.13 (0.85)	2.75 (0.94)	−2.81 **
Emotional neglect	172 (49)	3.05 (0.87)	2.58 (0.94)	−4.79 **
Physical neglect	179 (51)	3.07 (0.87)	2.54 (0.93)	−5.45 **
Loss of biological parent	109 (31)	3.12 (0.88)	2.67 (0.93)	−4.23 **
Domestic violence	113 (32)	3.23 (0.85)	2.61 (0.91)	−6.10 **
Household member substance abuse	126 (36)	3.17 (0.89)	2.61 (0.91)	−5.64 **
Household member mental illness	126 (36)	2.97(0.88)	2.72 (0.96)	−2.37 *
Household member incarcerated	73 (21)	3.07 (0.95)	2.74 (0.92)	−2.71 **

** *p* < 0.01, * *p* < 0.05.

**Table 3:** The association between ACEs and problematic media use.

	ΔR^2^	Β
**Step 1**	0.25	
Child age		−0.05
Child gender (1 = m, 2 = f)		−0.16 **
Child race (1 = White, 2 = other)		0.06
Length of time in foster care		0.30 **
Child weekly screen time		0.40 **
	F(5, 337) = 23.73 **	
**Step 2**	0.30	
Child age		−0.09
Child gender (1 = m, 2 = f)		−0.16 **
Child race (1 = White, 2 = other)		0.05
Length of time in foster care		0.21 **
Child weekly screen time		0.40 **
Total ACEs		0.24 **
	F(6, 336) = 25.06 **	

** *p* < 0.01.

## Data Availability

The data presented in this study are available on request from the corresponding author. The data are not publicly available due to participant confidentiality.
